# 60 Years of Progress—CDC and Infectious Diseases

**DOI:** 10.3201/eid1207.060531

**Published:** 2006-07

**Authors:** Tanja Popovic, Dixie E. Snider

**Affiliations:** * Centers for Disease Control and Prevention, Atlanta, Georgia, USA

**Keywords:** CDC, infectious diseases, history, commentary

Malaria Control in War Areas was formed in 1942 to ensure that the areas around military bases in the southern United States remained malaria-free. Initial facilities were modest, a few rooms on the sixth floor of the Volunteer Building on Peachtree Street in Atlanta. Hardly anyone could have foreseen the future of this small organization. But Joseph W. Mountin, who was charged with setting it up, was not just anyone. An architect of modern public health, Mountin quickly realized that malaria control operations serving the needs of the states (response to state calls for help, laboratory and epidemiologic investigations, training) could become the foundation for improving the health of the nation.

Indeed, in 1946 the Public Health Service established the Communicable Disease Center to work not only on malaria but on typhus and other infectious diseases. The following year, a token payment of $10 was made for a 15-acre area on Clifton Road to house the operations. In the next 60 years, minor changes were made to the name (Center for Disease Control, Centers for Disease Control, Centers for Disease Control and Prevention), but the initials, CDC, remained the same. The campus on Clifton Road grew to include 2 biosafety level 4 laboratories and other state-of-the-art facilities; operations were established in Morgantown, Cincinnati, Fort Collins, and overseas; and the work expanded to include all infectious diseases, as well as occupational health, toxic chemicals, injury, chronic diseases, health statistics, and birth defects.

A magnet for gifted scientists and other professionals looking to serve in public health, CDC has attracted an exceptional cadre of talent over the years. Mountin was succeeded by leaders who pushed the agency to new levels of achievement, constantly probing new challenges and seeking new public health solutions. The thousands who work in laboratories and offices or trot the globe on epidemiologic investigations; the physicians, veterinarians, microbiologists, statisticians, economists, social scientists, other scholars, and support personnel; the many volunteers who serve on institutional review and other boards and committees; and CDC's many partners in academia, industry, clinical practice, and state and local governments all share unequivocal dedication to public health.

In this climate of idealism and dedication, the achievements have been many and span all areas. CDC scientists, typically working with like-minded colleagues, identified and characterized several infectious agents and emerging infectious diseases; invented devices, tools, and stains for diagnoses and systems for surveillance; demonstrated the value of combining laboratory practices and epidemiology; and through vision and leadership, worked closely with state and local health departments to increase their effectiveness as public health organizations. Some in its midst made such major contributions that microorganisms were named after them (Lee Ajello, *Ajellomyces* spp.; Dannie Hollis, *Vibrio hollisiae*; Don Brenner, *Neisseria brenneri*; Robert Weaver, *Neisseria weaveri*; Joseph McDade, *Legionella micdadei*).

CDC led the US campaign to immunize all children against vaccine-preventable infectious diseases; efforts to "link" states in search of foodborne disease outbreak causes by using molecular approaches to trace the causative organisms (PulseNet); efforts to translate science to practice, protecting women and children from such emerging infection-related conditions as toxic shock syndrome and aspirin-associated Reye syndrome.

Achievements in international health have been major benchmarks. CDC contributions range from support for and leadership of the global effort to eradicate smallpox to the establishment of Projet SIDA in Africa to initiate scientific research on the HIV/AIDS epidemic.

**Figure 1:**
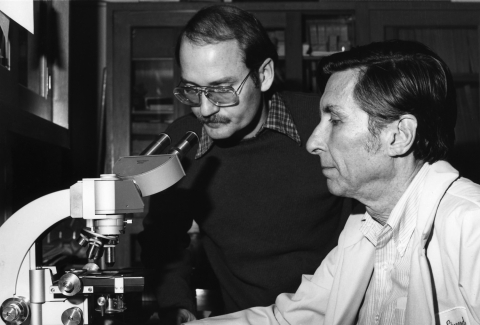
On January 14, 1977, the director of the Center for Disease Control's laboratory division, Charles C. Shepard, and microbiologist Joseph E. McDade isolated the agent that caused an outbreak of respiratory disease among members of the American Legion in July 1976. (Photo courtesy of Public Health Image Library, Centers for Disease Control and Prevention)

**Figure 2:**
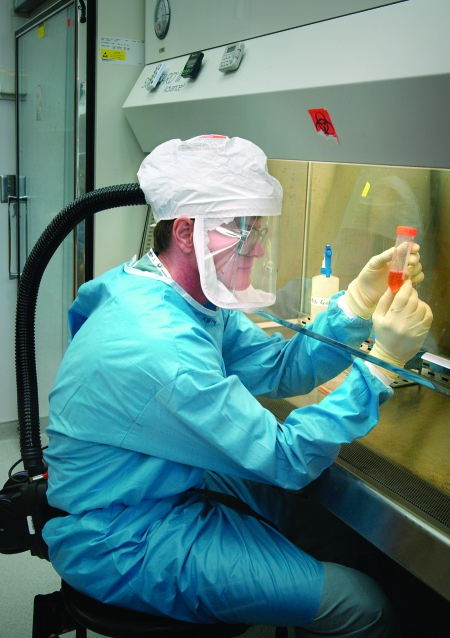
Terrence Tumpey recreated the 1918 influenza A (H1N1) virus to identify characteristics that made it such a deadly pathogen. (Photo by James Gathany, courtesy of Public Health Image Library, Centers for Disease Control and Prevention)

Science has changed in the past 60 years. Laboratory techniques used to detect, identify, and characterize microorganisms have moved from Petri dish and viral culture to real-time polymerase chain reaction and genome sequencing. During the 1976 Christmas holidays, a CDC laboratory scientist, using simple microbiologic methods, injected guinea pigs with material from persons who died of Legionnaires' disease. When some guinea pigs died, he injected their spleen into chicken eggs. He saw what was later confirmed to be the cause of this disease by looking under the light microscope (Figure 1). Thirty years later, others at CDC are able to identify all of almost 200,000 nucleotides that compose the genome of the smallpox virus. But science moves on. Recently, CDC scientists and colleagues have been able to recreate and reconstruct the 1918 influenza virus that caused the death of 40 to 50 million people (Figure 2). Information technology advances have enabled modeling to predict illness and death under specific circumstances, facilitate advance planning, and improve preparations for natural and human-made disasters.

Infectious diseases have changed in the past 60 years. All but hailed as being under control, they have found new virulence, emerging and reemerging globally without end. The new landscape of disease has required changes in management and control. The spectrum of science expertise has broadened, from entomologists and parasitologists (at Malaria Control in War Areas and the 1950s) to epidemiologists, microbiologists, and immunologists (predominating in the 1960s to 1980s). Over the past 2 decades, the CDC community has become increasingly multidisciplinary, embracing molecular biologists, geneticists, bioinformatics specialists, statisticians/mathematicians, behavioral and social scientists, modelers, economists, and other scholars.

What have not changed are the unique links between epidemiology and multiple other disciplines and between science and practice that keep CDC on the "speed dial" of every state and local public health official, every World Health Organization representative, and every minister of health worldwide. What has not changed is CDC's passion for science and public health. CDC scientists are proud to have served with so many colleagues and partners around the world on some of the greatest challenges to public health over the past 60 years. Nothing tells us we can rest on our collective laurels, impressive though they may be. Indeed, the most important lesson we have learned is that working together in research, applied public health, and preventive action is paramount because the emerging infectious disease and microbiologic challenges of the next 60 years may be even tougher than those we have already faced.

